# Assessing the population-wide exposure to lead pollution in Kabwe, Zambia: an econometric estimation based on survey data

**DOI:** 10.1038/s41598-020-71998-5

**Published:** 2020-09-15

**Authors:** Daichi Yamada, Masato Hiwatari, Peter Hangoma, Daiju Narita, Chrispin Mphuka, Bona Chitah, John Yabe, Shouta M. M. Nakayama, Hokuto Nakata, Kennedy Choongo, Mayumi Ishizuka

**Affiliations:** 1 Graduate School of Arts and Sciences, The University of Tokyo, Tokyo, 153-8902 Japan; 2 Faculty of Economics and Business, Hokkaido University, Sapporo, Hokkaido 060-0809 Japan; 3 School of Public Health, The University of Zambia, Lusaka, Zambia; 4 School of Humanity and Social Sciences, The University of Zambia, Lusaka, Zambia; 5 School of Veterinary Medicine, The University of Zambia, Lusaka, Zambia; 6 Faculty of Veterinary Medicine, Hokkaido University, Sapporo, Hokkaido 060-0818 Japan

**Keywords:** Environmental economics, Risk factors, Environmental sciences

## Abstract

This study quantitatively assessed the population-wide lead poisoning conditions in Kabwe, Zambia, a town with severe lead pollution. While existing data have reported concerning blood lead levels (BLLs) of residents in pollution hotspots, the data representing the entire population are lacking. Further, selection bias is a concern. Given the lack of compulsory testing schemes, BLLs have been observed from voluntary participants in blood sampling surveys, but such data can represent higher or lower BLLs than the population average because of factors simultaneously affecting participation and BLLs. To illustrate the lead poisoning conditions of the population, we expanded the focus of our surveys and then econometrically estimated the BLLs of individuals representing the population, including those not participating in blood sampling, using background geographic, demographic, and socioeconomic information. The estimated population mean BLL was 11.9 μg/dL (11.6–12.1, 95% CI), lower than existing data because of our wide focus and correction of selection bias. However, the scale of lead poisoning remained immense and 74.9% of residents had BLLs greater than 5 μg/dL, the standard reference level for lead poisoning. Our estimates provide a deeper understanding of the problem and a foundation for policy intervention designs.

## Introduction

Lead poisoning is one of the most serious and harmful consequences of environmental pollution. High levels of lead intake adversely affect the functioning of the circulatory and nervous systems, which can be fatal in extreme cases, whereas low-level exposure can also reduce cognitive ability and cause developmental disorders^1–4^. The adverse health effects can further result in poor school performance, lowered educational attainment and lifetime earning, and behavioural disorders^5–8^. While the use of lead in certain products, such as gasoline and paints, has been globally banned or reduced, lead poisoning remains imposing considerable costs to society, particularly in low- and middle-income countries, owing to the continued use of lead in various products, mining and smelting activities, and the lack of remediation for contaminated environments^4,9,10^.

Kabwe, Zambia, provides a devastating example of lead pollution. The fourth largest town with a population of approximately 200,000 as of 2010 was once the site of prominent lead and zinc mining activities, and the pollution problem has received attention since the 1970s^11^. Although the mine was formally closed in 1994, mining residues were abandoned in a dumping site adjacent to residential areas, locally known as Black Mountain, and continued to contaminate surrounding areas through the flow of wind and water^12–15^. Presently, Kabwe is listed as one of the ten most polluted sites in the world and the health conditions of the residents are concerning^16,17^. Lead poisoning is often measured by blood lead levels (BLLs). Recent standards, such as the one adopted by the Centers for Disease Control and Prevention (CDC) of the United States^18^, often regard 5 μg/dL as a reference level for lead poisoning, and chelation therapy is recommended for those with BLLs above 45 μg/dL. We also adopt these levels for reference—although health damages have been reported for BLLs below 5 μg/dL^1,4^, and we do not imply that BLLs below 5 μg/dL are safe. Previous studies on Kabwe have reported BLLs exceeding 45 μg/dL, including the levels normally considered fatal^15,19–22^. The scale of the problem is also large. According to the Toxic Sites Identification Program (TSIP)^23^, Kabwe’s lead contamination affects the largest number of people, with 120,000, among all the confirmed cases of lead contamination around the world.

Despite these alarming reports, representative data of the extent of lead poisoning among the entire population of Kabwe are lacking. This can be attributed to two issues related to data collection. First, most previous surveys have primarily focused on a small sample of data collected from children residing in areas around the mining sites. Although the pollution problem is the most acute in these areas, and children are generally at the highest risk of lead poisoning^4,18^, the data coverage has been far from representative. Second, while BLL data were obtained from those who voluntarily participated in studies, given the lack of public mechanisms for formal and compulsory blood lead testing, there could be selection bias in the data, a problem widely recognised in the medical, statistical, public health, and economics literature^24–28^. Data from voluntary (or self-selected) participants in studies generally can fail to reflect the conditions of the population if certain factors simultaneously affect participation decisions and the outcomes of interest. In the context of our study, the residents voluntarily participating in the blood sampling can be those particularly concerned about or suspected to have lead poisoning, and their BLLs can be lower or higher than the population average. Various background characteristics, including both observable (e.g. education, age, employment and living standards) and unobservable ones (e.g. health preferences), can also form their willingness and constraints to participate and directly affect BLLs. As the demographic composition, socioeconomic conditions and pollution levels are diverse in Kabwe, data reflecting these diversities and correcting for potential selection bias are essential.

The purpose of this study is to quantitatively assess the prevalence of lead poisoning among the entire population of Kabwe (administratively, the district of Kabwe). Our methodology to accomplish this purpose is twofold. First, we chose our sample individuals based on random sampling covering the entire Kabwe district. Although we could obtain BLL data from the subset of the chosen individuals who voluntarily participated in blood sampling, this expanded our focus beyond that of previous studies and helped define the target samples representing the population. Quick results of the blood sampling survey are available elsewhere^22^. Second, we employed econometric models to estimate BLLs for the representative sample individuals. Concurrently with the blood sampling survey, we conducted a household survey to obtain background socioeconomic, demographic, and geographic information for the sample individuals, including those who did not participate in the blood sampling survey. We then used these data to econometrically estimate the equation to determine BLLs and, finally, calculated the BLLs of the entire sample individuals. We paid attention to the potential differences in both observable and unobservable characteristics between the participants and non-participants under two econometric methods: ordinary least squares (OLS) and Heckman’s sample selection model^24^.

The contributions of our study are twofold. First, this study illustrates the severity and diversity of the lead pollution problem in Kabwe and help policymakers design remedial measures. This study is the first attempt to systematically obtain representative estimates of the lead poisoning conditions of residents in the entire Kabwe district. The estimated mean BLL was 11.9 μg/dL and 74.9% of residents had BLLs above the standard reference level of 5 μg/dL. Data representing the population are important to fully understand the pollution problem in Kabwe. Representative data can also serve as a foundation for a policy intervention design and cost–benefit analysis. Further, our estimates shed light on the extent of risks facing low- and middle-income countries, contributing to studies quantifying the global burden of general and pollution-related diseases^29–32^. Despite the substantial impact of lead poisoning on the global disease burden, there remain gaps in the literature, and existing data do not precisely depict the burden of diseases at contaminated sites^31^.

The second contribution is methodological and refers to the management of selection bias. Health surveys are often subject to selection bias analogous to our data, which can lead to biased conclusions^27,28^. The methodology adopted not only is consequential for bias mitigation in the current study. Although the details of our specifications would need modifications, our approach is applicable to many other cases where formal and compulsory testing for a disease is lacking.

## Methods

### Data collection and potential selection bias

We conducted two joint surveys from July to September 2017 in Kabwe: the Kabwe Household Socioeconomic Survey (KHSS) 2017 conducted by the Central Statistical Office of Zambia and University of Zambia under the supervision of the authors, and a BLL survey performed by the authors. The surveys were approved by the University of Zambia Research Ethics Committee (UNZAREC; REF. No. 012-04-16). Further approvals were granted by the Ministry of Health through the Zambia National Health Research Ethics Board and the Kabwe District Medical Office. The data were collected in accordance to the Declaration of Helsinki, and the informed consent was obtained from all the study participants including the parents/legal guardian of the minor subjects for participating in the study.

The two surveys were designed consistently and targeted the same sample households selected in the following two-step approach. In the first step, utilising the Zambia’s national census frame which divides the Kabwe district into 384 standard enumeration areas (SEAs), we randomly selected 40 SEAs across the entire district. In the second step, we randomly selected 25 households (and a few replacements) from each sampled SEA. The sampling weights were generated to account for population differences across the SEAs.

The KHSS 2017 conducted interviews with 895 households (4,900 individuals) at houses and collected data on socioeconomic, demographic and geographic information. The response rate was 88.2%, and we could regard the data adjusted by the sampling weights as representative of the entire Kabwe population (for more details of the survey, see the report^33^).

To obtain BLL data, we conducted a blood sampling survey concurrently with the KHSS 2017. For hygiene and ethical considerations, we selected 13 local clinics to perform the blood sampling, instead of collecting blood at houses. We invited up to four members (two children aged 10 years or younger and their parents or guardians) from each sample household for the blood sampling. We prioritised young children over children older than 10 years old. The invitations were made sequentially. We assigned identical venues and dates for households from the same SEAs. The typical assigned dates had a 3-day window from the day after the invitation. However, we allowed for some flexibility and sampled the blood of those who visited the clinic even after the assigned time window, as long as the clinic was operational for households from other SEAs. Therefore, the window for blood sampling was effectively the number of days from the day after the invitation until the pre-set blood sampling period in each clinic was over, which had a substantial variation across households from 3 days to a month. We revisit this feature of the survey window when setting up our econometric model later. A total of 372 households (41.6%) participated in the blood sampling and, on average, 2.2 members from the participating households provided blood samples.

We performed blood digestion and metal extraction as described by our previous study^34^ with minor modifications and measured BLLs using an Inductively Coupled Plasma-Mass Spectrometer (ICP-MS). In addition, we also measured BLLs with a portable analyser, LeadCare II, to obtain quick results^22^. However, we in this study focus on the ICP-MS data, considering their general accuracy. See the Supplementary Material Section S1 for details on the methods used to measure BLLs and the difference in the data between the two analysers.

Regardless of the accuracy of the techniques, however, we further need to account for the risk of selection bias in the BLL data. In the absence of formal and compulsory testing mechanisms, we relied on individuals’ voluntary (self-selected) visits to the clinics. However, the participants in blood sampling could have traits leading to higher or lower BLLs than the population. Such traits can include education, gender, age and living standards. The survey design did not prioritise children aged 11 years or older, and this could also contribute to the deviation of characteristics, although a small number of such children attended clinics. Moreover, certain unobservable characteristics affecting BLLs can further differ between the participants and non-participants. For example, those with greater preferences for health possibly had low BLLs but tended to participate in the blood sampling surveys, whereas those with a high innate physiological capacity for lead excretion possibly tended not to participate because they had low BLLs and did not perceive symptoms of lead poisoning. These issues can lead to selection bias, and the raw data observed from the voluntary participants can fail to illustrate the lead poisoning conditions of the population.

### BLL estimation approach

To correct for potential selection bias, we first estimated the equations to explain BLLs of children aged 0–10 years and adults aged 19 years or above. Then, using the estimated equations, we calculated BLLs for all individuals, including children aged 11–18 years and those in the other age groups who did not participate in the blood sampling.

BLLs generally depend on the ambient pollution level, the opportunities of exposure to pollution, the physiological capacity of lead absorption and excretion, and the knowledge and technologies used to prevent lead poisoning. We controlled for ambient pollution levels by including the distance, direction, and altitude of household location—the first two variables are with respect to the mine waste dumping site (Black Mountain). The remaining factors were measured by age and various other individual and household characteristics denoted by ${{\varvec{X}}}_{i}$. Data for these variables are available regardless of participation in blood sampling. We assumed the following equation for BLL:

1 $$\begin{aligned} \log BLL_{i} & = \beta_{dis} \log distance_{i} + \beta_{dir1} direction_{i} + \beta_{dir2} direction_{i}^{2} \\ & \quad
+ \beta_{alt} altitude_{i} + f\left( {age_{i} } \right) + {\varvec{X}}_{i} \user2{\gamma^{\prime}} + \varepsilon_{i}. \hfill \\ \end{aligned}$$

The logarithmic form for BLL adjusts its distribution to approximately normal—BLL is bounded from below and has a skewed distribution—and allows the factors on the right-hand side to have proportional effects rather than level effects. ${\varepsilon }_{i}$ is the independent and identically distributed error term that captures noise, such as casual fluctuations and measurement errors in BLLs, and the effects of unobservable factors. While we presented a single equation above, we assumed different equations for children aged 0–10 years and adults aged 19 years or above.

Below, we discuss our specification in detail.

### Geographic factors

Existing studies have examined the relationship between the geographic location and ambient pollution level^12–14^. Since lead is transported from the mine waste dumping site through the flow of wind and water, the distance from the site is negatively correlated with ambient lead levels. The soil lead contamination spreads to the western side of the site, particularly towards the west-northwest (WNW), which corresponds to the direction of the prevailing local wind. The contamination also slightly extends to the low-elevation south-eastern side, reflecting pollution transported by water. The northern and southern sides are the least contaminated.

We defined $distanc{e}_{i}$ as the distance between the mine waste dumping site and the location of $i$’s household, with ${\beta }_{dis}<0$ expected. Also, we assumed that the WNW is the most contaminated and, accordingly, we defined $directio{n}_{i}$ as the radian of the acute angle passing through WNW, the mine waste dumping site, and the location of $i$’s household. That is, the household location is WNW at $directio{n}_{i}=0$, either north-northeast or south-southwest at $\pi /2$, and east-southeast (ESE) at $\pi$. We employed a quadratic specification in Eq. (1), which allows BLLs to have two peaks at WNW and ESE if ${\beta }_{dir1}<0$, ${\beta }_{dir2}>0$ and $-{\beta }_{dir1}/\left(2{\beta }_{dir2}\right)<\pi$. We statistically assessed the appropriateness of the specification for direction in Supplementary Material Section S2. We also used altitude in metres, $altitud{e}_{i}$, considering that elevated areas can be less exposed to dust and water flows, although the general tendency of land elevation can be absorbed by the direction variables.

### Age and other covariates

For children, we assumed a non-linear relationship between their ages and BLLs and defined the following functional form:

2 $$f\left( {age_{i} } \right) = \left[ {\phi_{0} + \phi_{1} mage_{i} + \phi_{2} mage_{i}^{2} } \right] \times I\left( {age_{i} < 2} \right) + \phi_{3} age_{i} \times I\left( {age_{i} \ge 2} \right).$$

$I\left( \cdot \right)$ is an indicator function that takes the value of 1 if the argument condition is satisfied, and $mag{e}_{i}$ denotes age in months. The functional form reflects the findings in the literature. Young children are generally at a high risk of lead poisoning. Playing outside and age-appropriate hand-to-mouth behaviours expose them to lead, and their gastrointestinal absorption of lead is high^4^. Foetuses and infants born to exposed mothers absorb lead in utero and through breastfeeding^35^. Consequently, BLLs often reach a peak at or before the age of 24 months and then decrease as children grow older, reflecting their physical and behavioural growth^1,36^. Thus, we employed a specification that allows an inverted U-shaped relationship between the logarithmic BLL and age up to 23 months, but assume a linearly decreasing relationship between the two factors for children aged 2 years or above.

For adults, the physiological foundation of the BLL-age relationship is not clear, but age-related changes in metabolism and lifestyle can affect BLLs. We simply assumed a log linear relationship between BLL and age for adults.

In addition, we used the following individual and household characteristics, denoted as ${{\varvec{X}}}_{i}$, for children: a dummy variable for female; the mothers’ education level (grades), which reflects their general, health-related and lead-related knowledge; a dummy variable for children whose mothers were absent (the mothers’ education level was set at zero for such children); a dummy variable for female-headed households; household size; dependency ratio (the proportion of household members aged 0–15 years and 65 years or above); and the log of per capita household expenditure, which measures living standards. We also used dummy variables for household location: urban areas, small-scale farming areas, large-scale farming areas, and the Makululu compound—an area of informal settlement where public services are poorly delivered. We set urban area as the base category.

For adults, we continued to use the dummy variables for female and household location, household size and dependency ratio but dropped the variables related to mothers and household heads. The per capita household expenditure was not used, either, because it is not exogenous for adults. Instead, we used their own education level, which reflects living conditions to certain extent as well as knowledge levels. We also used a dummy variable for marital status, which takes the value of one for either married or co-habiting individuals, and the duration of residence in Kabwe (in years) to account for the effects of long-term lead exposure.

### Econometric methods to estimate BLL equation

We considered two methods to estimate Eq. (1). The first one is OLS, which directly estimates Eq. (1) from the data of the participants in the blood sampling survey. If the bias in BLLs are attributable to the difference in observable factors between the participants and non-participants, then the OLS estimate of Eq. (1) is unbiased and can be used to obtain estimates representing the population. However, as previously mentioned, unobservable characteristics can also affect both BLLs and participation decisions. This can disrupt the error term distribution and bias the OLS estimate of Eq. (1).

To account for this risk, we also adopted Heckman’s sample selection model^24^. This model corrects for the bias in unobservable factors by simultaneously estimating the probability of participation (selection equation) for the entire sample, including non-participants. Specifically, we considered the following selection equation:

3 $$\begin{aligned} \Pr \left( {i\;participates} \right) & =\Psi \{ \delta_{dis} \log distance_{i} + \delta_{dir1} direction_{i} + \delta_{dir2} direction_{i}^{2} \\ & \quad + \delta_{alt} altitude_{i} + g\left( {age_{i} } \right) + {\varvec{X}}_{i} \user2{\xi^{\prime}} + \zeta window_{i} \} , \\ \end{aligned}$$

where $\Psi$ is the normal distribution function with the probability density function of $\psi$, ${\varvec{X}}_{i}$ is the same as in Eq. (1), and $g\left( {age_{i} } \right)$ has the functional forms identical to $f\left( {age_{i} } \right)$. The bias in Eq. (1) can be fixed by estimating Eq. (1) with the inverse Mills ratio, $\psi /\Psi$.

In the sample selection model, the use of an exclusion restriction variable, which affects the probability of participation but not BLL, is preferable. We used the number of days of the blood sampling window denoted by $windo{w}_{i}$ as an exclusion restriction. As described above, the blood sampling window was effectively the number of days that the assigned clinic remained operational for blood sampling after the day following the invitation. Other factors being equal, households that received early invitations and had longer time windows would more easily manage to attend clinics and would have higher probabilities of participation. The exogenous nature of the blood sampling window renders it irrelevant for BLLs.

### Estimation of the representative BLLs

After obtaining the BLL equations, we estimated the BLLs of the representative sample individuals by inputting their characteristics on the right-hand side of the equations. We applied the survey’s sampling weights when aggregating the estimated BLLs.

To estimate the BLLs of adolescents aged 11–18 years, who were basically not covered in our BLL survey and thus not used in the BLL equation estimations, we used the equation for children aged 0–10 years, assuming that age-BLL trend, which we expected to be negative, would hold up to the age of 18 years.

Next, we calculated the number of the residents with BLLs above 5 μg/dL by interacting the estimated proportion of those with such BLLs and the total population. Considering the population growth, we used the population estimates of our own^33^ and the Central Statistical Office of Zambia^37^, both as of 2017, instead of 200,000 as of 2010.

Further, we present two graphical results. The first one is an in-depth examination of the mean BLLs across age groups. In the second one, we simulated the geographic variation of the mean BLLs. We divided the entire Kabwe district into 1 km × 1 km grids, and estimated the mean BLL in each grid cell. Distance and direction were measured for each cell and other independent variables were measured by the means in the ward—official inner-district division—to which the cell corresponds (we provide additional technical notes before showing results).

All estimations were performed using Stata 15 software.

## Results

### Observed BLL data

The observed mean BLL among the participants in the blood sampling survey was 15.9 μg/dL, in which we did not make econometric adjustments (Table 1). The 50 percentile (median) BLL was 11.3 μg/dL, indicating a skewed distribution. Male BLLs tended to be higher than female ones. BLLs were generally negatively associated with age. Overall, approximately 5.3% of the participants reported BLLs exceeding 45 μg/dL. This proportion was 14.2% among children aged 0–5 years, but only nine adults (2.0%) reported such high BLLs. The observation size for those aged 11–18 years were small.

**Table 1:** Observed blood lead levels (BLLs) of participants.

	All	Male	Female	0–5 years	6–10 years	11–18 years	19 years or above
**Panel A: BLLs (μg/dL)**							
Mean	15.9	17.5	14.7	22.9	21.0	14.6	11.3
(95% CI)	(14.9, 17.0)	(15.7, 19.2)	(13.4, 16.0)	(19.8, 26.0)	(18.7, 23.3)	(9.2, 20.0)	(10.4, 12.3)
25 percentile	4.6	5.3	3.9	5.9	6.8	5.3	3.6
50 percentile	11.3	11.9	11.0	18.5	20.7	10.2	9.2
75 percentile	22.4	24.2	20.0	31.6	30.1	23.8	15.2
**Panel B: percentage of those with the following BLLs**							
< 5 μg/dL	27.5	24.0	30.2	22.4	16.8	23.8	33.6
5–45 μg/dL	67.1	70.2	64.8	63.4	78.1	76.2	64.4
> 45 μg/dL	5.3	5.7	5.0	14.2	5.2	0.0	2.0
Observations	806	349	457	183	154	21	447

Based on surveys in Jul–Sep 2017.

*CI* confidence interval.

### Characteristics of blood sampling participants and non-participants

The characteristics of the participants and non-participants in blood sampling were not identical. Among children aged 0–10 years, the two groups significantly differed in terms of household location, size and living standards, with *P* values below 0.10 (Table 2). Among adults, the characteristics of the two groups were more clearly distinct, with *P* values mostly below 0.01 (Table 3). Therefore, the participants in blood sampling were not a random subset of our study target. Their BLLs (Table 1) can fail to represent the lead poisoning conditions of the population.

**Table 2:** Summary statistics for the characteristics of children aged 0–10 years.

	Participants	Non-participants	*P* value
	Mean ± SD	Mean ± SD	
Distance	6.25 ± 6.03	6.07 ± 5.31	0.60
Direction	1.12 ± 0.85	1.32 ± 0.85	< 0.01
Altitude	1,185 ± 9.78	1,185 ± 8.97	0.83
Age	5.16 ± 2.88	5.21 ± 3.20	0.81
Female	0.46 ± 0.50	0.49 ± 0.50	0.33
Household size	6.45 ± 2.57	6.98 ± 2.72	< 0.01
Dependency ratio	0.54 ± 0.14	0.54 ± 0.15	0.86
Mothers’ education	6.37 ± 4.36	6.39 ± 4.77	0.93
Mother absent	0.17 ± 0.37	0.19 ± 0.40	0.26
Female head	0.23 ± 0.42	0.22 ± 0.41	0.62
Household expenditure per capita^a^	414 ± 416	505 ± 777	0.04
Urban area	0.31 ± 0.46	0.36 ± 0.48	0.08
Small-scale farming area	0.27 ± 0.43	0.20 ± 0.40	0.08
Large-scale farming area	0.13 ± 0.34	0.19 ± 0.39	0.02
Makululu compound	0.31 ± 0.46	0.25 ± 0.43	0.02
Blood sampling window	11.2 ± 9.88	9.26 ± 8.36	< 0.01
Observations	338	1,176	

*P* values of *t* tests on the null hypothesis of identical mean.

^a^Monthly values in local currency, Zambian kwacha. Based on surveys in Jul–Sep 2017.

**Table 3:** Summary statistics for the characteristics of adults aged 19 years or above.

	Participants	Non-participants	*P* value
	Mean ± SD	Mean ± SD	
Distance	6.64 ± 6.36	5.23 ± 4.68	< 0.01
Direction	1.13 ± 0.81	1.38 ± 0.90	< 0.01
Altitude	1,185 ± 10.3	1,187 ± 8.65	0.01
Age	41.7 ± 14.8	35.6 ± 14.8	< 0.01
Female	0.64 ± 0.48	0.51 ± 0.50	< 0.01
Own education	8.02 ± 3.91	9.86 ± 4.06	< 0.01
Married	0.77 ± 0.42	0.51 ± 0.11	< 0.01
Duration of residence in Kabwe	26.2 ± 15.3	21.7 ± 13.8	< 0.01
Household size	5.68 ± 2.51	6.46 ± 2.94	< 0.01
Dependency ratio	0.46 ± 0.21	0.40 ± 0.21	< 0.01
Urban areas	0.31 ± 0.46	0.48 ± 0.11	< 0.01
Small-scale farming areas	0.27 ± 0.45	0.15 ± 0.35	< 0.01
Large-scale farming areas	0.13 ± 0.33	0.16 ± 0.37	0.07
Makululu compound	0.29 ± 0.46	0.22 ± 0.41	< 0.01
Blood sampling window	10.8 ± 9.33	9.29 ± 7.72	< 0.01
Observations	447	1,923	

*P* values of *t* tests on the null hypothesis of identical mean. Based on surveys in Jul–Sep 2017.

### Estimated BLL equation for children

In the BLL equation estimation based on OLS (Table 4, column I), the coefficients of the distance and direction variables had expected signs with *P* values below 0.01. BLL was decreasing in the distance, whereas the relationship between BLL and direction was U-shaped, with the highest peak at WNW, the lowest peaks at northeast and south ($directio{n}_{i}\approx 5\pi /8$), and a small peak at ESE. The explanatory powers of distance and direction were so large that R^2^ remained at 0.67 even after dropping other independent variables. Considering the strong powers of these factors and given that the values of these variables were similar among neighbouring households, we clustered standard errors for SEAs (in all the subsequent estimations as well). Altitude did not have a significant effect.

**Table 4:** Estimation results of the blood lead level (BLL) and selection equations for children aged 0–10 years.

	(I) OLS BLL		(II) Heckman selection		(III) Heckman BLL	
Log distance	− 0.755	(< 0.01)	− 0.0397	(0.78)	− 0.753	(< 0.01)
Direction	− 1.08	(< 0.01)	− 0.485	(0.13)	− 1.07	(< 0.01)
Direction squared	0.294	(< 0.01)	0.123	(0.21)	0.293	(< 0.01)
Altitude	0.0053	(0.44)	− 0.0038	(0.55)	0.0053	(0.41)
Being < 2 years old	− 2.23	(0.04)	− 1.48	(< 0.01)	− 2.21	(0.03)
Monthly age, < 2 years old	0.308	(0.05)	0.122	(0.07)	0.307	(0.03)
Monthly age squared, < 2 years old	− 0.0093	(0.07)	− 0.0037	(0.14)	− 0.0093	(0.05)
Yearly age, $\ge$2 years old	− 0.0507	(< 0.01)	− 0.0511	(< 0.01)	− 0.0500	(< 0.01)
Female	− 0.0188	(0.68)	− 0.0618	(0.42)	− 0.0179	(0.68)
Mothers’ education	− 0.0212	(0.10)	− 0.0007	(0.95)	− 0.0212	(0.09)
Mother absent	0.0145	(0.92)	− 0.144	(0.28)	0.0164	(0.90)
Female head	− 0.0226	(0.75)	0.0619	(0.54)	− 0.0241	(0.72)
Household size	− 0.0202	(0.11)	− 0.0464	(< 0.01)	− 0.0194	(0.08)
Dependency ratio	0.469	(0.07)	− 0.413	(0.24)	0.474	(0.06)
Log per capita household expenditure	0.0181	(0.75)	− 0.0438	(0.55)	0.0188	(0.73)
Large-scale farming area	− 0.0050	(0.99)	0.363	(0.14)	− 0.0101	(0.97)
Small-scale farming area	0.116	(0.59)	− 0.0107	(0.96)	0.115	(0.57)
Makululu compound	0.149	(0.26)	− 0.189	(0.37)	0.149	(0.24)
Blood sampling window			0.0202	(< 0.01)		
Inverse Mills ratio					− 0.0193	(0.90)
Constant	− 1.71	(0.84)	5.07	(0.50)	− 1.77	(0.82)
Observations	338		1,514			
R-squared	0.742					

*P* values are in parentheses and calculated based on standard errors clustered for the standard enumeration areas (SEAs), the survey’s primary sampling unit. Based on surveys in Jul–Sep 2017.

*OLS* ordinary least squares.

Age also had a significant effect. BLL peaked at 16.5 months, which is close to the average age of children to stop breastfeeding in Kabwe, 15.8 months^33^. This suggests a role of lead transfer through breastfeeding. BLL decreased by approximately 5% per year from the age of two years.

Among other factors, the dependency ratio raised BLLs, albeit with a marginally significant *P* value of 0.07. This suggests the possibility that parents in households with high dependency ratios failed to take sufficient precautionary measures for lead exposure. Mothers’ education reported a negative coefficient but its effect was insignificant with a *P* value of 0.10. Similarly, the per capita household expenditure did not have a significant coefficient.

Under Heckman’s sample selection model, the probability of participation significantly depended on age and household size (Table 4, column II). Although household income per capita reported significantly different means between the participants and non-participants (Table 2), its effect on participation was insignificant after other factors were controlled for. Conversely, while the mean age was almost identical between the two groups (Table 2), age had a significant non-linear effect on the probability of participation. The exclusion restriction, the duration of blood sampling window, had a significant effect with a *P* value below 0.01. However, the resulting BLL equation was similar to the OLS estimate (Table 4, column III). The inverse Mills ratio did not have a significant effect on BLLs with the *P* value of selection bias greater than 0.10. Therefore, selection bias was limited in terms of unobservable factors and the OLS estimate of the BLL equation was not significantly biased.

### Estimated BLL equation for adults

Under OLS, the effects of distance and direction were similar to those for children: BLL decreased with distance and had a U-shape relationship with direction, reaching the lowest levels in the northeast and south (Table 5, column I). Altitude had a negative coefficient, but was not significant with a *P* value above 0.10. Age and being female had significantly negative effects, although the marginal effect of age was moderate compared to that for children, approximately 0.5% per year. Own education also had a significantly negative effect on BLL, suggesting that knowledge or living conditions indicated by education levels affected adult BLLs. Duration of residence in Kabwe significantly increased BLLs.

**Table 5:** Estimation results of the blood lead level (BLL) and selection equation for adults aged 19 years or above.

	(I) OLS BLL		(II) Heckman selection		(III) Heckman BLL	
Log distance	− 0.926	(< 0.01)	− 0.0214	(0.91)	− 0.910	(< 0.01)
Direction	− 1.39	(< 0.01)	0.0134	(0.97)	− 1.40	(< 0.01)
Direction squared	0.386	(< 0.01)	− 0.0484	(0.68)	0.399	(< 0.01)
Altitude	− 0.0074	(0.11)	− 0.0017	(0.83)	− 0.0072	(0.09)
Age	− 0.0047	(0.02)	0.0085	(< 0.01)	− 0.0062	(0.01)
Female	− 0.245	(< 0.01)	0.388	(< 0.01)	− 0.307	(< 0.01)
Own education	− 0.0200	(0.01)	− 0.0296	(< 0.01)	− 0.0155	(0.06)
Married	0.0650	(0.25)	0.617	(< 0.01)	− 0.0384	(0.63)
Duration of residence	0.0042	(0.04)	0.0058	(0.04)	0.0033	(0.11)
Household size	0.0000	(0.99)	− 0.0702	(< 0.01)	0.0112	(0.35)
Dependency ratio	0.121	(0.28)	0.401	(0.03)	0.0492	(0.68)
Large-scale farming area	0.516	(0.01)	0.523	(0.13)	0.436	(0.01)
Small-scale farming area	0.470	(< 0.01)	0.0157	(0.95)	0.465	(< 0.01)
Makululu compound	− 0.160	(0.07)	− 0.0286	(0.85)	− 0.184	(0.07)
Blood sampling window			0.0176	(0.01)		
Inverse Mills ratio					− 0.214	(0.15)
Constant	13.2	(0.02)	0.416	(0.97)	13.3	(0.01)
Observations	447		2,370			
R-squared	0.697					

*P* values are in parentheses and calculated based on standard errors clustered for the standard enumeration areas (SEAs), the survey’s primary sampling unit. Based on surveys in Jul–Sep 2017.

*OLS* ordinary least squares.

The remaining columns show the results under Heckman’s sample selection model. The participation decisions of adults depended on various individual and household characteristics. Those with high levels of education and from large households were less likely to participate, whereas older adults, women, and those either married or co-habiting, having resided in Kabwe for a prolonged period, and from households with high dependency ratios were more likely to participate. The duration of the blood sampling window significantly increased the probability of participation. However, similar to the results for children, the inverse Mills ratio did not have a significant effect with *P* value above 0.10.

### Representative estimates of lead poisoning conditions

We estimated the BLLs of 4,898 individuals, all but two sample individuals who had missing information, that represent the lead poisoning conditions of the entire population (Table 6). Since the selection bias in terms of unobservable factors was not significantly observed (Tables 4, 5), we used the BLL equations obtained under OLS. All figures hereafter were weighted by the survey’s population weights.

**Table 6:** Estimated blood lead levels (BLLs) representative of Kabwe population.

	All	Male	Female	0–5 years	6–10 years	11–18 years	19 years or above
**Panel A: BLLs (μg/dL)**							
Mean	11.9	12.6	11.2	18.2	15.4	11.2	8.9
(95% CI)	(11.6, 12.1)	(12.2, 13.0)	(10.8, 11.5)	(17.3, 19.2)	(14.7, 16.1)	(10.7, 11.6)	(8.6, 9.1)
25 percentile	5.0	5.5	4.6	7.9	6.8	4.9	4.2
50 percentile	8.7	9.5	7.9	12.7	11.8	8.2	6.8
75 percentile	16.1	17.4	14.9	28.1	24.2	17.1	12.0
**Panel B: percentage of those with the following BLLs**							
< 5 μg/dL	25.1	21.5	28.6	9.6	9.8	26.0	34.9
5–45 μg/dL	74.1	77.7	70.5	85.8	89.9	74.0	65.1
> 45 μg/dL	0.8	0.8	0.8	4.6	0.3	0.0	0.0
Observations	4,898	2,380	2,518	768	746	1,014	2,370

The survey’s population weights were applied. Based on surveys in Jul–Sep 2017.

*CI* confidence interval.

The representative mean BLL was 11.9 μg/dL, with a 95% confidence interval of 11.6–12.1 μg/dL, which is 2.4 times higher than the standard reference level of 5 μg/dL. 74.9% of the residents had BLLs above 5 μg/dL. This proportion, as of 2017, corresponds to approximately 202,500 individuals based on our population estimate, 270,389 individuals^33^, and to approximately 170,400 individuals based on the relatively moderate population projection of 227,551 individuals by the Central Statistical Office of Zambia^37^. The 50 percentile (median) was 8.7 μg/dL. Men had significantly higher BLLs than women (the *P* value for zero difference is below 0.01). Notably, only 9.6% of children aged 0–5 years and 9.8% of children aged 6–10 years had BLLs below 5 μg/dL, although this study expanded the focus beyond the immediate neighbourhood of the mine waste dumping site. 4.6% of children aged 0–5 years had BLLs above 45 μg/dL, but our estimates did not predict such high BLLs for adolescents aged 11–18 years and adults.

**Figure 1:**
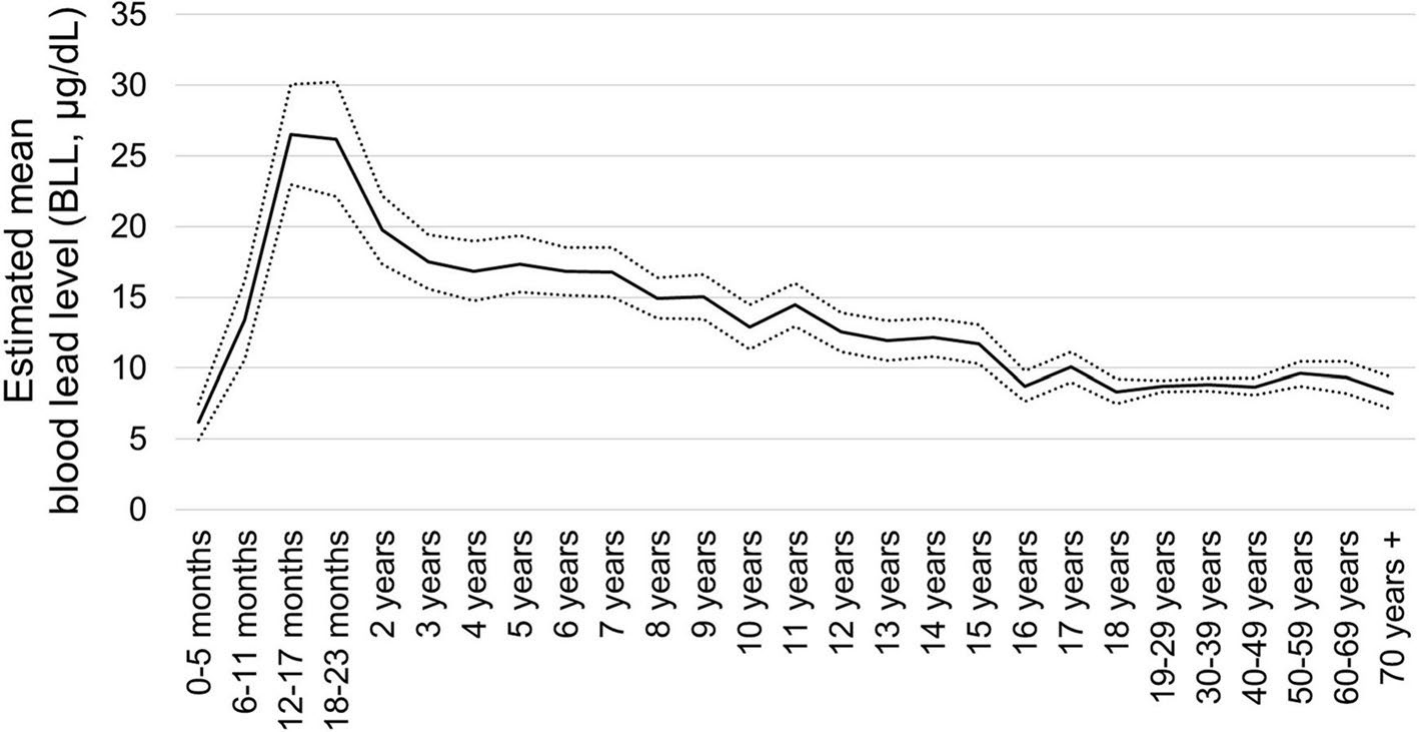
Estimated blood lead levels (BLLs) and age. Solid line: mean. Dotted lines: 95% confidence interval. Based on surveys in Jul–Sep 2017.

Figure 1 depicts the in-depth relationship between the estimated BLLs and age. After peaking within the ages of 12–23 months, BLLs for children demonstrated a declining trend with age, albeit with fluctuations. Note that the BLLs of those aged 18 years and 19–29 years were continuously connected. This suggests that we successfully estimated the BLLs of those aged 11–18 years from the equation for children aged 0–10 years.

**Figure 2:**
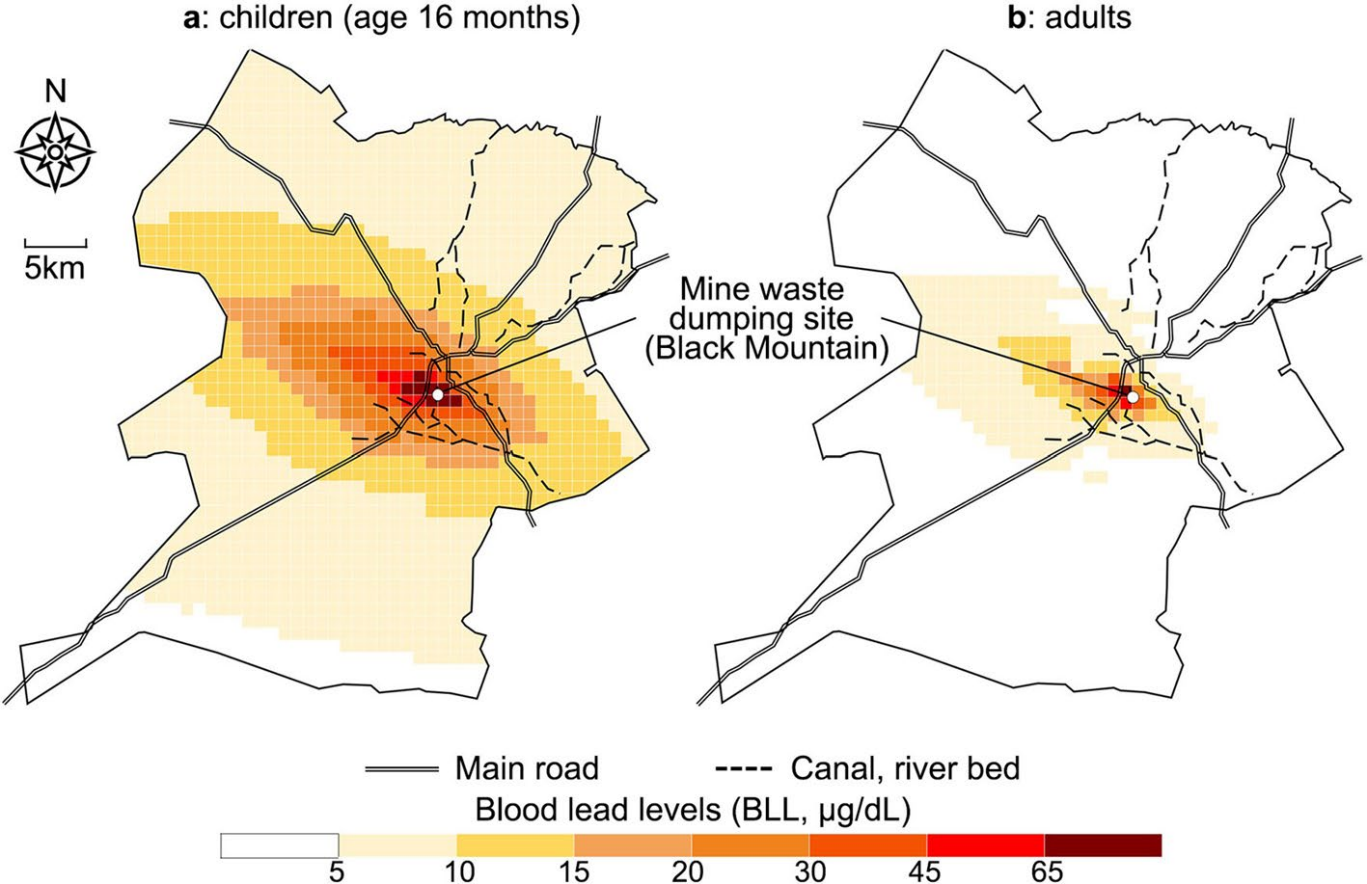
Geographic distribution of estimated blood lead levels (BLLs). (**a**) Children (age 16 months). (**b)** Adults. Based on surveys in Jul–Sep 2017.

Figure 2 illustrates the simulated geographic distributions of BLLs, separately for children (a) and adults (b). To obtain the figure for children, we set age at 16 months, when BLL reaches the maximum. Thus, the figure for children can be considered the geographic distribution of the maximum BLL that a child with average traits is expected to report. Age is set at the local mean for adults, approximately 34–38 years.

For both children and adults, BLLs were high in WNW and ESE. BLLs greater than 45 μg/dL were found in the neighbourhood of the mine waste dumping site. BLLs tended to decrease with distance. However, the BLLs of children, at the maximum, exceeded 5 μg/dL throughout most areas.

## Discussion

This study estimated BLLs representative of the lead poisoning conditions among the entire population of Kabwe, Zambia, using the combined dataset of the ICP-MS measures of BLLs and a socioeconomic household survey. As in the previous studies on Kabwe and other health surveys in general, we were faced with the risk of selection bias in the BLL data in terms of both observable and unobservable factors, owing to non-random participation in the blood sampling survey. To overcome this problem, we employed econometric methods that controlled for differences in observable and unobservable factors between participants and non-participants in the blood sampling survey.

Our estimates showed that the mean BLL for the population was 11.9 μg/dL (Table 6), which is 25.2% lower than the mean of the observed BLLs of the participants (15.9 μg/dL, Table 1). While unobservable factors reported a minor bias (Tables 4, 5), the observable factors were not identical between the participants and non-participants (Tables 2, 3). In particular, the participants (or their parents) tended to have lower education levels and resided in Kabwe for a prolonged period, which were factors positively associated with BLLs. The age composition and household location were also different. These differences led to higher BLLs among the participants. Our estimate of the mean BLL was also lower than the ones in existing studies^15,19–21^, mainly because their focus was placed mostly on pollution hotspots, but their data could be faced by selection bias similar to our observed data. Further, both our estimated and observed mean BLLs were lower than our early results based on LeadCare II analyser^22^. Although LeadCare II analyser is considered fairly accurate, our samples included higher BLLs than ones to which LeadCare II is often applied, and this apparently led to overestimation of BLLs (see the Supplementary Material Section S2).

Nevertheless, our results illustrate the devastating lead poisoning problems in Kabwe. We confirmed critically high BLLs among children residing in the most contaminated areas. Further, the mean BLL of our estimates was considerably higher than the standard reference level of 5 μg/dL, and the proportion of those with BLLs above this level amounted to 74.9%. Based on our population estimate as of 2017^33^, this proportion corresponds to 202,500 individuals (or 170,400 based on another population estimate^37^), which is greater than an existing estimate of 120,000 in the TSIP^23^.

These estimates provide a foundation for policy intervention designs. Since lead poisoning was widespread across the entire Kabwe district, interventions that span across the entire population are required. Thus, although immediate interventions, such as chelation therapy proposed under a World Bank project^38^, could focus on pollution hotspots, interventions to reduce lead transportation, such as capping the mine waste dumping site with concrete or clean soil, would be of fundamental importance. Our estimates also provide grounds for proper cost–benefit evaluations of interventions. For large-scale interventions, the benefits for the entire population, not only the residents in hotspots, need to be accounted for, and this requires population-level data. Proper cost–benefit evaluations are important for sustainability of interventions as they require large costs and long-term commitment (e.g. monitoring and maintenance).

Our methodology has an implication for other cases of health studies. Medical and clinical data collected through voluntary participation in testing can be subject to analogous selection bias problems to ours, particularly in cases in which formal and compulsory testing schemes are lacking. The extent of disruptions caused by selection bias can vary by case, and our econometric specifications would require modifications if applied to other cases. Nevertheless, the principle of our approach—collection of background data from the representative sample individuals, including those who did not participate in the medical testing, and correction of deviation in the characteristics of the participants—is applicable to various cases in which selection bias is a concern.

Finally we address the limitations of our study. First, our methodology was not employed to perfectly predict the BLL of each individual. Our estimates reflected variations of BLLs by gender, age groups, areas within Kabwe and various other factors but did not fully reflect idiosyncratic variations. Certain individuals with particular idiosyncratic factors can have high or low BLLs even if their traits and residential locations are associated with low or high BLLs. The second limitation, related to the first one, is the general difficulty to econometrically predict extreme outcomes. Such outcomes are scarce and idiosyncratic factors prevail over systematic ones. In our case, a small proportion of adults did report such BLLs, but our estimates did not predict such BLLs for adults. Finally, while we employed BLL as the measure, a comparison with alternative measures would improve the understandings of the lead poisoning problem in Kabwe. For example, bone and tooth conditions would reflect the effects of long-term lead exposure better, and clinical conditions would reflect idiosyncratic variations in the sensitivity to lead intake. Analysing these alternative measures could be the topic for further research.

## Supplementary information

Supplementary Information.

## Data Availability

The datasets used in the current study are not publicly available based on the ethical approvals from the University of Zambia Research Ethics Committee, the Ministry of Health of Zambia and the Kabwe District Medical Office.
