# Supplementary material for: Assessing the population-wide exposure to lead pollution in Kabwe, Zambia: an econometric estimation based on survey data
Source: Sci Rep. 2020 Sep 15;10:15092. doi: 10.1038/s41598-020-71998-5 (PMC7492281; doi:10.1038/s41598-020-71998-5)
Supplement: Supplementary file 1 — Supplementary Information. [file 41598_2020_71998_MOESM1_ESM.pdf]

Supplements of

Assessing the population-wide exposure to lead pollution in Kabwe, Zambia: an econometric estimation based on survey data

Daichi Yamada[1*], Masato Hiwatari[2], Peter Hangoma[3], Daiju Narita[1], Chrispin Mphuka[4], Bona Chitah[4], John Yabe[5], Shouta M. M. Nakayama[6], Hokuto Nakata[6], Kennedy Choongo[5], Mayumi Ishizuka[6]

[1] Graduate School of Arts and Sciences, the University of Tokyo, Tokyo, 153-8902, Japan.

[2] Faculty of Economics and Business, Hokkaido University, Sapporo, Hokkaido, 060-0809, Japan.

[3] School of Public Health, the University of Zambia, Lusaka, Zambia.

[4] School of Humanity and Social Sciences, the University of Zambia, Lusaka, Zambia.

[5] School of Veterinary Medicine, the University of Zambia, Lusaka, Zambia.

[6] Faculty of Veterinary Medicine, Hokkaido University, Sapporo, Hokkaido, 060-0818, Japan.

[*] Correspondence: dyamada@global.c.u-tokyo.ac.jp.

S1. Notes on BLLs measured by ICP-MS

The blood samples were examined at the authors' laboratory at Hokkaido University, Japan, instead of local clinics. The blood samples were sealed and frozen immediately after collected, and exported under a permission by the Ministry of Health of Zambia.

Blood digestion and metal extraction were performed as described by [1] with minor modifications. All laboratory materials and instruments used in metal extraction were washed in 2% nitric acid (HNO_3) and rinsed at least twice with distilled water. 200 µL of whole blood was placed in pre-washed digestion vessels, followed by acid digestion using 5 mL of two-fold diluted ultrapure nitric acid (Cica reagent, Specific gravity of 1.38, 60%; Kanto Chemical Corp., Tokyo, Japan) and 1 mL of ultrapure hydrogen peroxide (Cica reagent, 30%; Kanto Chemical Corp.). The digestion and metal extraction were operated using microwave digestion system (Speed Wave MWS-2; Berghof, Eningen, Germany) following the manufacturer's instruction. After cooling, extracted solutions were transferred into 15-mL plastic tubes and diluted to a final volume of 10 mL with double distilled deionized water (Milli-Q; Millipore, Bedford, MA).

BLLs were determined using ICP-MS (7700 Series; Agilent Technologies, Tokyo, Japan). Analytical quality control was performed using the certified reference material of Seronorm Trace Elements Whole Blood L-2 (Sero, Billingstad, Norway). Replicate analysis of

these reference materials showed good recoveries (95–105%). The instrument detection limit was 0.001 µg/L.

In addition to the ICP-MS measures, we used LeadCare II to obtain quick results, which are available in [2]. Generally, LeadCare II is considered fairly accurate although its inaccuracy has also been pointed out [3]. In our case, the BLLs measured in these two analysers turned out considerably different. The observed mean BLL in the LeadCare II data, before econometric adjustments, was 21.7 µg/dL (n=827, 95% confidence interval (CI) of 20.2–23.2 µg/dL), 36.5% higher than the ICP-MS mean of 15.9 µg/dL (n=806, 95% CI of 14.9–17.0 µg/dL, the main text Table 1). Although the two measures were similar up to 20 µg/dL, LeadCare II tended to provide higher BLLs above that level. Also, after applying the econometric model, the estimated population mean BLL was 15.6 µg/dL based on LeadCare II (n=4,898, 95% CI of 15.2–16.0 µg/dL), 31.1% higher than the estimated population mean based on ICP-MS data, 11.9 µg/dL (n=4,898, 95% CI of 11.6–12.1 µg/dL, the main text Table 6). With such differences, considering the general accuracy of ICP-MS, we focused on the ICP-MS data in the main text.

Note that our early results in [2] have a larger sample size than the observed data in the current study, since our early results included additional individuals outside of the random sampling framework. However, since we aim to obtain the estimates representing the population, we in the current study focused on individuals chosen under the random sampling (further, socioeconomic survey data were collected only for the individuals chosen under random sampling). The sample sizes of the ICP-MS and LeadCare II means above are also slightly different, because of the difficulty to collect the sufficient amount of blood from infants under 12 months old to be analysed by ICP-MS. However, the loss of such infants would not bias our estimated results as our econometric methods correct for the age composition of the samples.

S2. On the specification of the direction variables

In our estimations, we set the base direction to WNW and employed a quadratic form for the BLL-direction relationship. To check the appropriateness of our specification, we estimated equation (1) in the main text without the direction variables and plotted the residuals and the quadratic fitted curves (Supplementary Figure). The residuals reached the maximum between west and northwest for both children and adults, and the fitted curves appear to follow the residual patterns. Furthermore, as an alternative form, we re-estimated equation (1) in the main text by dropping $direction_i^2$ (i.e. assuming a linear relationship between BLLs and direction). Then R^2 decreased from 0.742 to 0.719 for children and from 0.697 to 0.659 for adults. This also supports the quadratic specification.

References

1. Nakata, H. et al. Reliability of stable Pb isotopes to identify Pb sources and verifying biological fractionation of Pb isotopes in goats and chickens. *Environ. Pollut.* **208**, 395–403 (2016).
2. Yabe, J. et al. Current trends of blood lead levels, distribution patterns and exposure variations among household members in Kabwe, Zambia. *Chemosphere* **243**, article number 125412 (2020).
3. Centers for Disease Control and Prevention. *Emergency preparedness and response potential for falsely low blood lead test results from LeadCare analyzers*. https://emergency.cdc.gov/han/han00403.asp (2018).

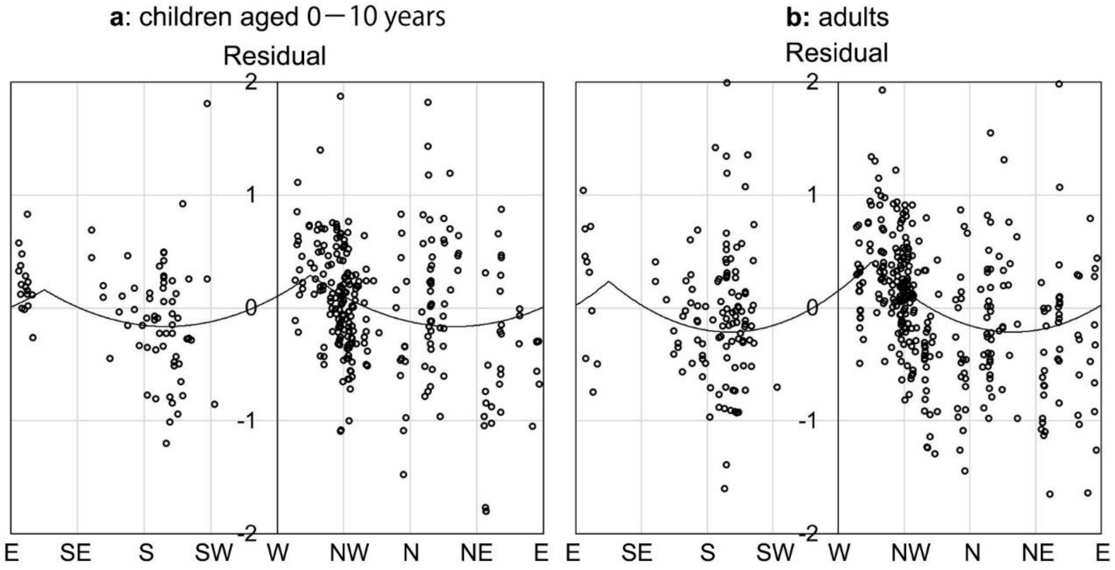

Supplementary Figure. The residual plots with respect to direction after regressions without direction. **a**: children aged 0–10 years. **b**: adults.
